# Chemical Modification Methods for Inulin- and Agavin-Type Fructans: Synthesis, Characterization, and Biofunctional Activity: A Review

**DOI:** 10.3390/molecules30132672

**Published:** 2025-06-20

**Authors:** Dafne I. Díaz-Ramos, Maribel Jiménez-Fernández, Oscar García-Barradas, Rosa Isela Ortiz-Basurto, Benoit Fouconnier

**Affiliations:** 1 Centro de Investigación y Desarrollo en Alimentos, Universidad Veracruzana, Xalapa 91190, Veracruz, Mexico; diazmc06@gmail.com; 2 Facultad de Ciencias Químicas, Universidad Veracruzana, Coatzacoalcos 96538, Veracruz, Mexico; 3 Instituto de Química Aplicada, Universidad Veracruzana, Xalapa 91190, Veracruz, Mexico; osgarcia@uv.mx; 4 Laboratorio Integral de Investigación en Alimentos, TecNM-Instituto Tecnológico de Tepic, Tepic 63175, Nayarit, Mexico; riobasurt@ittepic.edu.mx

**Keywords:** fructans, modification, chemical methods, structure, functionality

## Abstract

Inulin and agavin fructans have been widely used in the food industry as fat substitutes, wall materials, and prebiotics, among other applications. Chemical modifications offer several advantages, from enhancing functional properties to broadening industrial applications, making them a key area of research in biotechnology, nutrition, and food science. This review examines the chemical modifications of fructans, specifically the inulin and agavin types. It describes the most commonly used methods, their characteristics, and their impact on the physicochemical, functional, and prebiotic properties of fructans. Additionally, it explores the interactions underlying these changes. Modifications enhance, extend, or generate new biological properties and activities. While most yield positive outcomes, challenges remain, including a deeper understanding of the structure–bioactivity relationships and further toxicity assessments, particularly in agavins. These insights aim to guide future research and innovation in the field.

## 1. Introduction

Fructans, particularly those derived from inulin and agavins, are polysaccharides primarily composed of fructose units, sometimes terminating with a glucose residue. Due to their structural diversity and broad range of functional properties, they have received considerable attention in the scientific community. However, it is important to present their characteristics objectively and within a comparative context.

**Figure 1:**
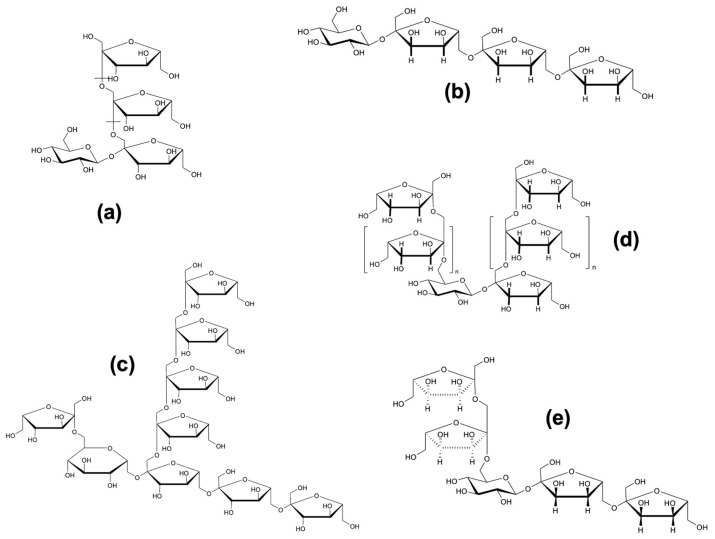
Molecular structures of inulin-type fructans (**a**), levans (**b**), graminans (**c**), inulin neoseries (**d**), levan neoseries (**e**) [15].

Structurally, fructans are linear or branched polymers in which β-(2→1) and/or β-(2→6) glycosidic linkages connect the fructose monomers [1]. They are classified based on their degree of polymerization (DP): chains with DP < 10 are referred to as fructooligosaccharides (FOS), while those with higher DP values are considered long-chain fructans. This classification is relevant because the DP significantly influences their physicochemical behavior and biological activity [2,3,4]. Fructan biosynthesis begins with a sucrose molecule, to which fructose residues are sequentially added through β-(2→1) and/or β-(2→6) linkages. As a result, fructans are also categorized as soluble dietary fibers with prebiotic potential. Based on the configuration of their glycosidic bonds and structural complexity, fructans can be subdivided into five major types: (a) linear inulin-type fructans, composed of β-(2→1)-fructofuranosyl linkages; (b) levan-type fructans, featuring β-(2→6) linkages, found in both plants and bacteria; (c) mixed-type fructans, such as those from grasses or agave (agavins), containing both β-(2→1) and β-(2→6) linkages; (d) inulin neoseries, characterized by a glucose residue positioned between two β-(2→1)-linked fructofuranosyl units; (e) levan neoseries, in which β-(2→1) and β-(2→6) fructofuranosyl units are located at both ends of a central sucrose core [5] (Figure 1. Molecular structures of fructans). This classification highlights the structural complexity and functional versatility of fructans, underscoring the importance of accurate terminology and precise biochemical description in scientific literature.

Fructans occur naturally in a wide variety of organisms and are predominantly obtained from plant sources, including *Cichorium intybus* (chicory), various monocots such as members of the Asparagales order, *Lolium* spp., and *Agave* spp. (e.g., *Agave tequilana*). They are also found in tubers of *Helianthus tuberosus* (Jerusalem artichoke) [6]. Importantly, certain bacterial species, particularly those from the genera *Bacillus* and *Zymomonas*, also synthesize fructans such as levans, which have demonstrated both industrial and biological relevance due to their distinct molecular structures and physicochemical properties [7]. The origin of fructans, whether plant-based or microbial, determines their degree of polymerization, branching patterns, and glycosidic linkages, which in turn influence their functional performance in food and pharmaceutical and agricultural applications.

Speaking specifically about inulins and agavins, they exhibit marked differences in both structure and degree of polymerization (DP), which significantly influence their chemical reactivity and potential applications. Inulin, which consists of linear chains of fructose units with β(2→1) linkages and a terminal glucose, generally exhibits a low to moderate DP (ranging from 2 to 60). Inulins with a DP greater than 20 are poorly soluble in water and possess amphiphilic characteristics, enabling them to form stable, three-dimensional microcrystalline gel networks at concentrations between 13 and 50%. Consequently, they are used as sugar substitutes due to their low caloric value and as fat replacers in foods, as they provide creamy textures similar to those of fat. These properties make inulin suitable for use in spreads, dressings, dairy products, baked goods, and ice cream, particularly in the formulation of low-fat or fat-free products [8]. In contrast, agave fructans possess a highly branched structure, incorporating both β-(2→1) and β-(2→6) linkages, with glucose residues located within the polymer backbone. Their DP can reach up to 32 or higher in mature agave plants [9]. This complex structure results in high water solubility, which makes agavins particularly useful in beverages, high-moisture products, or as stabilizing agents, especially for protein protection [6,10].

In addition, fructan structures feature a high number of hydroxyl (OH) groups (Figure 1), which confer a high hydrophilic character, limiting their stability and applications in food or pharmaceutical formulations. Therefore, the chemical modification of inulin and agavins has received considerable attention in recent scientific literature due to its positive impact on functional properties, stability, and industrial applications. These transformations allow the adjustment of key characteristics, such as solubility, hygroscopicity, viscosity, gelling capacity, and thermal stability [11,12]. Furthermore, they can enhance biological activities such as antioxidant, anti-inflammatory, prebiotic, and anticancer effects by inducing structural changes that generate or improve the accessibility of reactive functional groups [13]. Furthermore, chemical modifications significantly expand the versatility of polysaccharides, promoting their use as sustainable alternatives to synthetic polymers, particularly in the development of biodegradable packaging and active coatings [14]. In this context, it is essential to highlight the wide range of applications derived from the chemical modification of these fructans. This area represents a promising field of research, due to its potential in multiple industries, such as food, pharmaceuticals, and biomedicine.

Despite the significant benefits of chemical modifications, they also entail significant challenges, such as achieving controlled and targeted modifications without compromising the structural integrity of fructans. The reactions can generate products with highly heterogeneous functional group distribution, which affects reproducibility and functionality [16]. Modifications sometimes require aggressive reaction conditions (extreme pH, organic solvents, high temperatures), which can affect the fructan structure or limit its industrial viability for safety or sustainability reasons [17,18,19]. Scaling up these reactions to industrial processes remains a challenge, as it is complicated both economically and environmentally. It requires the design of processes compatible with continuous production technologies and environmental regulations [20,21]. Finally, food or biomedical applications require exhaustive studies on the toxicity, biocompatibility, and metabolic effects of the modified products, as well as compliance with international regulations [22,23]. However, these challenges underscore the need to continue developing more efficient, selective, safe, and environmentally friendly modification strategies that allow us to fully exploit the potential of fructans as functional materials in various industries.

In line with the above, this article provides an overview of the role and potential of modified fructans, particularly agavin and inulin, highlighting the opportunities they present in various scientific and industrial fields. The article begins with an overview of existing structural modification methods, followed by a review of the chemical methods described for agavins and inulins. The chemical characterization methods for these modifications and their effects on physicochemical, functional, and biological properties are then discussed. Finally, the challenges and prospects presented by these modified fructans are addressed.

## 2. Fructans Structure Modification Methods: A Description of Chemical Approaches

Modification can be achieved through physical, biological, chemical, and genetic methods or a combination thereof [24,25]. Physical methods include heat, microwaves, ultrasound, and high pressure, among others [26]. They have the advantage of being cost-effective compared to other methods. They are also easy to implement and are environmentally friendly, as they avoid the use of chemical reagents, resulting in less environmental pollution. However, they are energy-inefficient; for example, microwave-assisted extraction techniques can cause high localized temperatures, which could degrade the polysaccharides [27]. They also have limited scalability and fail to achieve specific structural changes in the polysaccharides [26]. Biological processes use enzymes or microorganisms [28]. These methods exhibit high specificity because enzymes can act on specific bonds, producing precise modifications. Furthermore, the reaction conditions are mild, and the use of biodegradable catalysts reduces environmental impact. Unwanted side reactions are minimized, preserving the integrity of the polysaccharide. However, enzymes and microbial cultures can be expensive to produce and maintain. As some enzymes are highly specific, their applicability to certain polysaccharides is limited. They have slower reaction times compared to chemical methods, which impacts yield [29,30]. Chemical methods, on the other hand, employ chemical reagents to change the structure and conformation of polysaccharides [24]. Among the most common chemical methods are sulfation, phosphorylation, carboxymethylation, methylation, and acetylation [31]. These methods allow a wide range of modifications to adapt functional properties. They possess greater bioactivity by significantly improving biological activities, such as antioxidant and antitumor effects [13,32,33]. On the other hand, the use of toxic reagents can generate hazardous waste, making disposal difficult and leading to safety issues. They also require extensive purification, which increases processing time and cost. Finally, genetic engineering modifies polysaccharide-producing bacteria to increase their production, modify substituents, or generate new polysaccharides. The modification groups affect the rheological behavior and biological activity of the polysaccharides [34,35,36]. Genetic methods offer the advantages of enabling the design of organisms to synthesize polysaccharides with specific structures and functions. They can be cultured to continuously produce polysaccharides. Furthermore, they facilitate the creation of new polysaccharides with unique properties for specialized applications. In turn, genetically modified organisms face strict regulations, especially regarding their use in food and pharmaceuticals. They require advanced knowledge in molecular biology and genetic engineering techniques. Furthermore, ethical considerations may limit their acceptance and commercialization [37,38]. In this context, chemical modifications of inulin- and agavin-type fructans have been reported to demonstrate enhanced functional properties and certain biological activities compared to their native forms. These improvements have enabled their application across various industrial sectors. The chemical modification of these fructans has emerged as a strategy to tailor their structural characteristics, enhance their functionality, and broaden their industrial relevance [39,40]. Based on recent publications, the most common chemical modification methods are outlined below, along with a discussion of their underlying reaction mechanisms, as well as their respective advantages and disadvantages.

### 2.1. Esterification

The modification of fructans through esterification involves the reaction of saccharide hydroxyl groups with acylating agents, leading to the formation of acetylated, benzylated, sulfated, and phosphorylated derivatives [41]. This strategy has been employed to tailor the physicochemical and functional properties of these polysaccharides for various industrial applications.

Díaz-Ramos et al. [42] demonstrated the effectiveness of this approach by modifying three different agave fructan fractions: native (NAF), high performance (HPAF), and high degree of polymerization (HDPAF) via lauroylation using lauroyl chloride in an alkaline NaOH medium. The resulting materials exhibited a high degree of substitution (DS = 2.36), which translated into enhanced swelling capacity, oil retention, and the introduction of new functional properties such as emulsifying and foaming abilities. Moreover, the modified fructans displayed improved thermal stability, making them suitable for use as emulsifiers, food additives, pharmaceutical excipients, and cosmetic ingredients. Subsequent studies have expanded on this work by incorporating lauroyl and palmitoyl groups into agave fructans using various acyl chlorides, achieving degrees of substitution ranging from 0.18 to 2.75 [43]. These modifications were designed to modulate the hydrophobicity of the material and to facilitate the controlled, sustained release of active compounds such as ibuprofen. The resulting derivatives were shown to possess high stability under gastric pH conditions and the ability to provide prolonged drug release, highlighting their potential as drug delivery vehicles. In a similar context, Ignot-Guitierrez et al. [44] reported that lauroyl chloride-based acylation enhanced the functional performance of fructans, indicating their suitability for inclusion in food systems to improve technological functionality. Additionally, inulin has been successfully modified using long-chain fatty acyl chlorides (C12, C14, C16, C18) in aqueous NaOH media to yield amphiphilic molecules. These derivatives demonstrated the capacity to form both oil-in-water and water-in-oil emulsions, further supporting their applicability in food, cosmetic, and pharmaceutical formulations [45,46].

An important operational advantage of these acylation processes is that they are typically carried out at room temperature, with heating applied only during the drying phase of the final product. In some cases, the reaction temperature ranges between 25 °C and 60 °C, which helps preserve the integrity of the polysaccharide by minimizing degradation or unwanted side reactions [42,44,46,47,48]. From a mechanistic perspective, these acylation reactions—particularly those involving acyl chlorides—proceed via a nucleophilic acyl substitution mechanism. In this pathway, the hydroxyl group (–OH) of the polysaccharide acts as a nucleophile, attacking the electrophilic carbon atom of the acyl chloride. This results in the displacement of a chloride ion (Cl^−^) and the formation of an ester linkage (R–COOR′). The reaction is favored in basic environments (e.g., NaOH), which promote deprotonation of the hydroxyl group and enhance its nucleophilicity [49]. This reaction pathway offers several notable advantages: it is chemically reproducible, especially under mild alkaline conditions. It enables the controlled modulation of key properties such as hydrophobicity, solubility, and thermal stability. It is typically conducted at low temperatures, reducing the risk of polymer backbone degradation. However, certain limitations must also be considered: the formation of byproducts (e.g., HCl) requires effective neutralization or removal to prevent adverse effects on the material’s functionality. The use of acyl chlorides demands careful handling due to their high reactivity and potential toxicity. The degree of substitution may be influenced by variables such as the solubility of the acylating agent and the three-dimensional structure of the fructans, requiring careful reaction optimization [39].

In summary, despite these challenges, esterification remains one of the most straightforward, versatile, and widely applied methods for the chemical modification of biopolymers, offering a flexible platform for tailoring their functional properties for diverse applications.

#### 2.1.1. Acetylation

Acetylation is an esterification reaction in which hydroxyl groups (–OH) present along the backbone of a polysaccharide—such as fructans (inulin and agavins)—are replaced by acetyl groups (–COCH_3_). This modification is typically performed using acetic anhydride or acetyl chloride, in the presence of a catalytic base such as sodium acetate or sodium hydroxide, and is often performed in polar solvents such as *N*,*N*-dimethylformamide (DMF) [17]. The introduction of these functional groups alters the spatial conformation of the biopolymer, significantly modifying its hydrophobicity, solubility, and other functional properties. Multiple studies have shown that the effect of acetylation depends on the degree of substitution (DS) achieved. A moderate increase in DS can lead to notable improvements, such as enhanced emulsifying capacity, which results from the introduction of hydrophobic domains that promote the formation of stable interfacial layers [41,50,51,52]. However, this modification also presents certain limitations. In some cases, acetylation may lead to a reduction in antioxidant activity, likely due to the partial degradation of the base polymer. Furthermore, the increase in hydrophobic groups results in decreased water solubility, which can limit their application in aqueous formulations. Therefore, precise control over the degree of substitution is essential to optimize the functionality without compromising other critical properties of the material [17].

Consistently, it has been demonstrated that the acetylation of agave fructans enhances their prebiotic effect and improves the survival of *Lactobacillus paracasei* during simulated gastrointestinal digestion [17]. Improvements in prebiotic activity and changes in the thermal properties of acetylated agave fructans have also been observed during fermentation with *Saccharomyces boulardii* [53]. Acetylated agave fructans have enabled efficient and prolonged drug release, demonstrating high stability at gastric pH when formulated into hydrophobic microspheres [43]. On the other hand, acetylated inulin has been evaluated as an encapsulation material for colon-targeted drug delivery of mesalamine, showing reduced enzymatic degradation by the colonic microbiota, which may enhance therapeutic efficacy [54]. Furthermore, inulin acetates (INAs) have demonstrated potential activity against pathogenic microorganisms. INAs with a degree of polymerization (DP) of 7–12 showed stronger antimicrobial effects, likely due to enhanced diffusion and greater interaction with fungal cells. In contrast, INAs with a DP of 22 exhibited lower bioactivity, which may be attributed to reduced mobility and solubility in the agar medium used during testing. These findings suggest that INAs could be applied as bioproducts against plant pathogens and in environmentally friendly formulations [55].

#### 2.1.2. Succinylation

Succinylation is a chemical modification process through esterification, in which the hydroxyl groups (–OH) present along the fructan backbone react with succinic anhydride, forming ester bonds that introduce additional carboxyl groups (–COOH) into the polysaccharide structure. This reaction can be carried out in the presence of polar organic solvents, such as *N*,*N*-dimethylformamide (DMF), and under controlled pH conditions, allowing an efficient and targeted modification [56,57]. From a structural perspective, succinylation can alter both the hydrophobicity and hydrophilicity of the biopolymer, depending on the nature of the anhydride used. For instance, the use of dodecenyl succinic anhydride introduces hydrocarbon chains that increase the hydrophobic character of the modified fructans. In contrast, the direct incorporation of non-aliphatic succinyl groups adds polar carboxylic functionalities, thereby enhancing the water solubility of the polymer and its ability to interact with charged surfaces [58]. Recently, studies have reported succinylation reactions involving both agave fructans (Agavinas) and inulins. Regarding agave fructan fractions, Díaz-Ramos et al. [17] reported their prebiotic effect, antibacterial properties, and ability to support the survival of *L. paracasei* in simulated gastric conditions. Notably, agave fructans with a high polymerization degree fraction enhanced probiotic survival during simulated gastrointestinal tests compared to unmodified fructans. Succinylation enhanced the antibacterial activity of the samples tested compared to their unmodified counterparts. Due to these promising results, agave fructans were considered for the formulation of prebiotic, symbiotic, and antiseptic products.

In a similar manner, Han et al. [59] synthesized three types of octenyl-succinylated (OSA) inulins, H25, DS2, and XL, corresponding to low (2–8), medium (2–18), and high (20–23) degrees of polymerization (DP), respectively. To evaluate their ability to encapsulate and release β-carotene upon lyophilization, the authors reported that inulin-OSA formed micellar aggregates in aqueous solution above a critical aggregation concentration (CAC) of 0.07% and demonstrated that β-carotene could readily dissolve into the hydrophobic cores of micellar aggregate cells. In terms of encapsulation, the β-carotene loading capacity was also higher in samples with higher DP, reaching up to 25 mg/g in XL, compared to 12 mg/g for H25 P (low DP). This is attributed to the greater number of hydrophobic segments distributed in long chains, which generates a more effective micellar core to host nonpolar compounds [45,60]. The particles obtained by lyophilization showed selective release under simulated intestinal conditions (pH 7.0), with no release under simulated gastric conditions (pH 2.5). These results suggest a potential application of inulin-OSA in the encapsulation, dissolution, and targeted delivery of hydrophobic drugs for nutraceutical, pharmaceutical, functional food, and medical applications.

Similarly, octenyl-succinylated inulin derivatives (OSA-inulin) were synthetized using inulin with different molar masses, and their ability to encapsulate the anticancer drug doxorubicin (DOX) was demonstrated. However, DOX solubilization was only achieved using OSA-inulins that formed micellar aggregates in aqueous solution above their critical concentration [61]. Additionally, the authors reported that, compared to free DOX, in vitro administration of OSA-inulin-DOX micelles significantly enhanced the growth inhibition of MCF-7 breast cancer cells. A faster cellular uptake rate was observed, indicating that the micelles were rapidly absorbed by the cells. These findings suggest that OSA-inulin holds great potential for encapsulation, dissolution, and targeted delivery of hydrophobic drugs, particularly for antitumor drugs.

### 2.2. Crosslinking

Crosslinking involves the formation of chemical side bonds between different chains and interactions with hydrogen bonds, reducing solubility and adhesion while enhancing the swelling properties of polysaccharides [62]. Although research on fructan crosslinking is limited, the synthesis and characterization of an inulin hydrogel crosslinked with pyromellitic dianhydride (PMDA) has been investigated. Three formulations with different amounts of PMDA (IN93HY1, IN93HY2, IN93HY3) were evaluated to study the effect of the degree of crosslinking. The IN93HY1 hydrogel reached a swelling of 1540%, qualifying it as a superabsorbent material, which is comparable with other smart hydrogels based on polysaccharides, such as guar gum or modified pectin [63,64]. The crosslinker content affected the crosslinking density and the molar mass between crosslinking points (Mc), decreasing from 390.6 g/mol (IN93HY1) to 152.39 g/mol (IN93HY3). This behavior has been reported by other authors as an indicator of denser and stiffer networks that reduce swelling and modulate the drug release rate [65]. Therefore, by varying the proportion of PMDA, hydrogels with different crosslinking densities can be obtained, which influences their swelling capacity as a function of pH. Furthermore, thermal analysis revealed that the hydrogels remained stable at physiological temperature (37 °C), which is sufficient for oral drug delivery applications. The novel pH-responsive hydrogel is considered promising for colon-targeted delivery since dianhydrides offer efficient crosslinking due to their rapid reactivity with the hydroxyl groups of polysaccharides [66]. To the best of our knowledge, no research has yet explored crosslinking in agave fructans.

### 2.3. Carboxymethylation

Carboxymethylation is one of the most widely employed chemical strategies for polysaccharide derivatization due to its simplicity, low cost, and minimal toxicity [67]. This process involves the etherification reaction between the hydroxyl groups of the polysaccharide and monochloroacetic acid (MCA), leading to the introduction of carboxymethyl groups (–CH_2_COOH) into the polymer backbone. The resulting derivatives often exhibit improved solubility and enhanced biological activity across a wide pH range [68]. From a mechanistic standpoint, the reaction proceeds via nucleophilic substitution, where the hydroxyl group of the polysaccharide attacks the electrophilic carbon in monochloroacetic acid, replacing the chlorine atom and forming a carboxymethyl ether linkage. This reaction typically occurs under alkaline conditions, which facilitate the deprotonation of the hydroxyl groups, enhancing their nucleophilicity [69]. In a pioneering study, Castañeda-Salazar et al. [70] reported the carboxymethylation of agave fructans using MCA in an alkaline medium. The resulting derivatives exhibited increases in both melting and decomposition temperatures, as well as an enhanced ability to bind water molecules, leading to significantly greater solubility without altering their core functional properties. Importantly, these modified fructans also demonstrated improved antimicrobial activity against Gram-negative bacteria such as *Escherichia coli* and *Salmonella enterica*. However, this remains the only published study to date specifically addressing carboxymethylated agave fructans, highlighting a critical gap in the literature. On the other hand, inulin has been extensively studied for carboxymethylation, often incorporating additional functional groups to enhance bioactivity. Mi et al. [71] synthesized five carboxymethyl inulin (CMI) derivatives by coupling the polysaccharide with heterocyclic compounds—namely 2APCMI, 3APCMI, 4APCMI, 2ATCMI, and 3ATCMI. Structural analysis confirmed the successful integration of pyridine, thiazole, and triazole groups. Among these, derivatives containing thiazole (2ATCMI) and aminopyridine (4APCMI) showed the highest biological activity, attributed to the –N=C–S– group and the para-positioned nitrogen in the pyridine ring, which enhanced molecular reactivity while minimizing steric hindrance [72,73]. In addition to exhibiting strong antifungal activity, these derivatives demonstrated notable antioxidant properties, validating the approach of combining heterocyclic rings with carboxymethylated backbones to improve membrane interaction and permeability [72,74]. Overall, these CMI derivatives displayed superior biocompatibility, elevated bioactivity, and low cytotoxicity, establishing a foundation for the development of novel antioxidants and antifungal agents applicable to medical, cosmetic, and pharmaceutical fields. Further studies explored the synthesis of carboxymethyl inulin derivatives containing various functional salts, aimed at evaluating antioxidant and antibacterial activity in vitro. Ten CMI derivatives were assessed, incorporating compounds such as thiosemicarbazide, aminoguanidine, aniline, and chlorinated anilines. Derivatives with thiosemicarbazide salts were particularly effective, achieving free radical scavenging rates up to 93.98% at 0.8 mg/mL, comparable to vitamin C as a positive control. Notably, CMI derivatives with thiosemicarbazide and aminoguanidine salts exhibited high antioxidant potential, which was attributed to electronegative nitrogen atoms and conjugated bonds, which serve as electron donors to neutralize free radicals. Furthermore, those containing thiosemicarbazide and aniline salts demonstrated antibacterial activity against both E. coli and Staphylococcus aureus, likely due to electrostatic interactions with the negatively charged bacterial membrane, leading to increased permeability and cell lysis [69]. Across all cases, the derivatives exhibited low cytotoxicity, reinforcing their potential in medical and food-related applications. Carboxymethylation offers several well-documented advantages: significantly improved aqueous solubility, especially over a wide pH range [70]; enhanced antioxidant and antimicrobial activity [69]; and low-cost reagents and operation under mild, nontoxic conditions. However, some limitations remain: the degree of substitution (DS) is typically moderate (between 0.13 and 0.48), which can limit the full potential for functional improvements. Strict pH control is essential during synthesis to avoid overreactions, degradation, or side reactions that could compromise the polymer’s structure or bioactivity [70].

### 2.4. Sulfation

Polysaccharide sulfation is a chemical modification that involves the introduction of sulfate groups (–OSO_3_^−^) into the hydroxyl groups of the polymer chain, typically using agents such as sulfuryl chloride or sulfuric acid in organic media. This process leads to the sulfonation of polysaccharide chains, generating polyanions with altered structures that can result in significant changes in biological activity [75]. Sulfate groups can be incorporated at various positions within the polysaccharide backbone, directly influencing the polymer’s physicochemical and functional properties. It has been demonstrated that sulfation markedly enhances the bioactivity of polysaccharides, particularly their antitumor activity [75]. Consequently, sulfation has been applied to the modification of inulin-type fructans, aiming to enhance their therapeutic and functional potential. For example, purified inulin from Jerusalem artichoke (P-JAP) was subjected to sulfation to evaluate its biological activity against HepG2 tumor cells (human liver cancer). It was modified by the sulfur trioxide–pyridine complex (SO_3_-Py), obtaining a degree of substitution (DS) of 0.56. The sulfated inulin derivative (S-JAP) exhibited greater antiproliferative activity against HepG2 cells compared to unmodified P-JAP. Furthermore, S-JAP promoted cell apoptosis and showed significantly higher inhibition rates than P-JAP in a dose- and time-dependent manner. At a concentration of 0.5 mg/L, the inhibition was 24.07% (24 h), 28.06% (48 h), and 31.16% (72 h). These findings suggest that sulfated inulin from Jerusalem artichoke has potential as an antitumor agent [76]. However, no studies evaluating sulfation in agave fructans have been reported to date.

### 2.5. Schiff Base Reactions

One of the emerging strategies for the functionalization of fructans is their modification through Schiff bases, a class of compounds that have gained attention due to their structural versatility and bioactive potential. The term *Schiff bases* originates from the German chemist Hugo Schiff, who in 1864 first described these compounds as the products of a condensation reaction between a primary amine and a carbonyl compound (aldehyde or ketone), forming a characteristic imine linkage (–C=N) [77,78,79]. From a chemical standpoint, the carbon–nitrogen double bond (C=N) introduces distinctive reactivity: the nitrogen atom acts as a base and nucleophile, donating electron pairs, while the carbon of the imine group is electrophilic, making it susceptible to nucleophilic attack. This dual nature enables Schiff bases to undergo various substitution and addition reactions and, in particular, to form stable complexes with transition metals, which is highly valuable in catalytic, medical, and analytical applications [80,81,82]. Among the advantages of this reaction is its ability to proceed under mild conditions, typically in aqueous or alcoholic media, without the need for toxic catalysts—making it compatible with green chemistry principles. Additionally, biopolymers modified with Schiff bases exhibit enhanced functional properties, including antimicrobial, antifungal, antioxidant, and anticancer activities, due to the presence of the imine group and its metal-complexing ability. They also display self-healing behavior, attributed to the reversible nature of the imine bond, which is particularly useful in biomedical and tissue engineering applications. However, this strategy also presents certain limitations. The stability of the imine linkage can be compromised in aqueous environments or under extreme pH conditions. Moreover, the biopolymer often requires pre-activation (e.g., oxidation to introduce carbonyl groups) prior to modification, adding complexity to the process. Although Schiff base formation is straightforward at the laboratory scale, scalability and reproducibility may pose challenges for the industrial production of modified materials [83,84,85].

In this context, inulin derivatives have been synthesized via Schiff base reactions to enhance their biological activity. Six distinct derivatives were prepared through aza-Wittig-type reactions with aromatic aldehydes, with or without phenolic hydroxyl groups, and their antioxidant and antifungal properties were evaluated against three phytopathogenic fungi. The results demonstrated a significant improvement in biological activity compared to unmodified inulin, highlighting their promising potential as biomaterials with excellent bioactivity and biocompatibility [80]. To date, no studies have reported the application of this modification in agave fructans, making it a promising area for future research in the fields of biotechnology, functional foods, and pharmaceutical development.

As described in this section, fructans, such as inulin and agavins, can be chemically modified through various strategies that allow the adjustment of their structural and functional properties according to specific application requirements. Each modification method offers distinct advantages and presents certain limitations, making the careful selection of the most appropriate approach essential, depending on the intended purpose.

For instance, if the objective is to enhance interaction with lipophilic compounds, increase gastrointestinal resistance, or improve antimicrobial activity, acylation methods such as acetylation or lauroylation are recommended, depending on the desired degree of hydrophobicity [17,42]. For applications requiring high solubility in aqueous media, compatibility with biological systems, and significant antioxidant activity, carboxymethylation is a suitable alternative, provided that the hygroscopicity of the resulting material is properly controlled [69,70]. Sulfation, on the other hand, is particularly indicated for the development of therapeutic biomaterials, such as anticoagulant or immunomodulatory agents, although its implementation requires caution due to the use of highly reactive reagents [76]. In contrast, modification through Schiff bases represents a more sophisticated and versatile approach, which is ideal for the design of intelligent functional materials with advanced pharmaceutical, nutraceutical, and biomedical applications, due to their ability to introduce specific functional groups with high bioactivity. Finally, succinylation offers various functional enhancements to modified fructans [17]. The introduction of carboxyl groups promotes interactions with cations and proteins, facilitating the formation of stable colloidal structures, such as gels or emulsions, which are especially useful in food and pharmaceutical matrices [59,61]. Additionally, increased resistance to gastrointestinal conditions has been reported, which enhances the survival of probiotic strains, along with significant antibacterial activity, which in some cases exceeds that observed in acetylated derivatives [42].

In general, the main differences between inulin and agavin focus on their molecular structure and branching, which directly affect their physicochemical properties and functional behavior. On the one hand, inulin forms gels and has been used as a fat substitute. Meanwhile, agavin stands out for its high solubility, low hygroscopicity, and complex structure, making it a versatile alternative for liquid applications and functional beverages [6]. These differences influence the chemical modification potential of each fructan. The linear nature of inulin facilitates targeted chemical derivatization, with consistent results. Agavins, due to their complex and heterogeneous structure, pose challenges for targeted modifications, resulting in broad substitution patterns and variable physicochemical outcomes. Knowing and understanding these differences is essential for selecting the ideal fructans for modification and application according to the research focus. Table 1 and Table 2 present a summary of the aforementioned studies on inulin and agavin, emphasizing their effects and applications.

**Figure 2:**
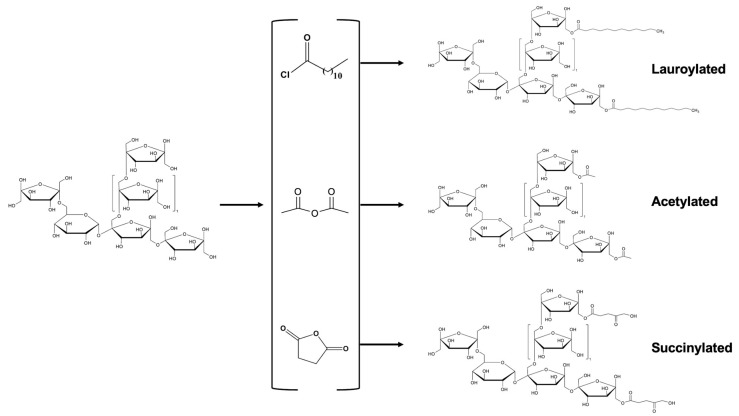
General reaction scheme of the functional groups incorporated into the fructan structure.

**Figure 3:**
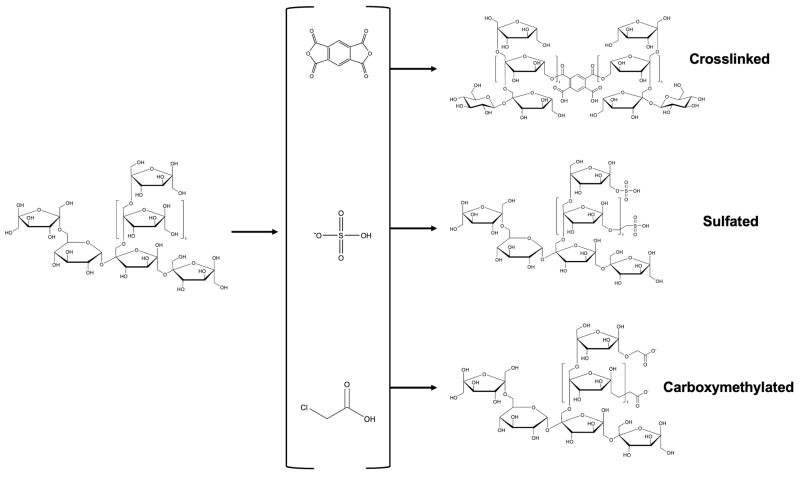
General reaction scheme of the functional groups incorporated into the fructan structure.

Each type of modification leads to a structural alteration involving the incorporation of various functional groups into the polysaccharide chain. In the case of fructans, groups such as acetyl, lauroyl, succinyl, carboxymethyl, and sulfate have been introduced (Figure 2 and Figure 3). These transformations not only modify the molecular structure of the chains but also significantly influence their biological and functional properties. As a result, changes in bioactivity and behavior are observed in the modified fructans. However, to ensure the effectiveness of these modifications, it is essential to conduct a detailed structural characterization, as outlined in the following section.

## 3. Structural Characterization of Modified Fructans

The modification of polysaccharides leads to the alteration of their structure [86]. However, this process can sometimes cause significant degradation of the polysaccharide. Therefore, it is crucial to characterize the structure modifications using chemical and physical methods to ensure that the intended polysaccharide is obtained. Accordingly, the modified fructans have been analyzed using FTIR, NMR, and XRD techniques to confirm the success of the substitution.

### 3.1. FTIR Analysis

The most representative FTIR signals obtained for each successful structural modification performed on agave fructans and inulin from the studies analyzed above are summarized in Table 3. In esterification reactions, which include acetylation, succinylation, and crosslinking, the most characteristic functional group in both agavins and inulin is the carbonyl group (C=O). This group is an indicator of ester bond formation and typically appears in the absorption range of 1700–1750 cm^−1^, as consistently reported in multiple studies. However, deviations from this range have been observed. For example, Díaz-Ramos et al. [42] and Ignot-Gutiérrez et al. [44] reported carbonyl stretching signals at lower frequency values. These shifts were attributed to hydrogen bond rearrangements and nonpolar interactions caused by the presence of long fatty acid chains, which modify the vibrational environment of the ester bonds [87]. In addition to the carbonyl signals, signals corresponding to methyl and methylene groups (–CH_3_ and –CH_2_–) were also consistently detected in esterified fructans, confirming the incorporation of aliphatic chains. In the case of crosslinking reactions, such as those involving pyromellitic dianhydride, an additional signal appears in the FTIR spectrum, which is attributed to the aromatic ring of the pyromellitic group. This signal serves as a key spectral marker that distinguishes these systems from simple esterifications. For carboxymethylation, the representative functional group is the carboxylic acid group (–COOH). The position and intensity of this signal can vary depending on the type of fructans (agavin vs. inulin), likely due to differences in molecular weight and degree of branching, which affect molecular interactions and, consequently, vibrational frequencies. In sulfation reactions, the presence of sulfate groups (–OSO_3_^−^) is confirmed by distinctive FTIR signals, along with characteristic vibrations of the sugar ring. A notable absorption band is observed around 3430 cm^−1^, attributed to sulfated hydroxyl (OH) groups, indicating successful substitution [59,88]. Finally, in inulin derivatives obtained through Schiff base reactions, FTIR spectra reveal signals corresponding to benzene rings, as well as acetyl, azide, and imine groups, depending on the specific structure of the derivative formed [80]. These diverse signals reflect the complexity and versatility of Schiff base chemistry applied to fructan functionalization.

### 3.2. NMR Analysis

NMR analysis provides detailed structural insights into modified polysaccharides [89]. This technique has proven highly effective in identifying the structural changes induced by fructan modifications. Depending on the monosaccharide composition and the specific research goals, one-dimensional NMR spectra (^1^H or ^13^C) are sometimes sufficient to detect relevant chemical shifts. However, two-dimensional NMR techniques, such as ^13^C–^1^H and ^1^H–^1^H correlation spectroscopy, are often required for more precise elucidation of molecular interactions and substitutions [31]. The structural modifications observed in fructans before and after chemical functionalization help explain subsequent changes in their biological, physicochemical, and functional properties. Table 4 compiles the most representative NMR signals reported in the studies analyzed in Section 2. Notably, ^1^H NMR analysis of both agavin and inulin revealed signals corresponding to methyl (–CH_3_) and methylene (–CH_2_–) groups. These proton resonances were detected in specific regions depending on the modification type:

- 0.80–2.65 ppm for esterification;
- 1.20–2.03 ppm for acetylation;
- 1.26–1.94 ppm for succinylation;
- 4.51 ppm for carboxymethylation.

These variations indicate that the type of fructans, the nature of the introduced functional group, and the degree of polymerization (DP) collectively influence the chemical environment, thereby shifting the corresponding NMR signals. In the case of Schiff base reactions, additional peaks related to acetyl groups, benzene rings, and other aromatic systems were identified, confirming the successful conjugation of aromatic functionalities.

### 3.3. Diffraction Analysis 

The X-ray diffraction technique provides insight into the conformation of polysaccharide chains [90]. It is often used to assess structural changes in polysaccharides following modification. The crystallinity of polysaccharides is governed by interactions between polymer chains, which influence their packing and organization. The blending or modification of polysaccharides allows changing crystallinity to enhance the properties and performance of polysaccharides [91]. Chemical modifications introduce functional groups into the polysaccharide backbone, significantly altering its crystallinity and morphology [47]. Surface modifications affect functional groups on the polysaccharide surface, while bulk modifications influence the entire structure [92]. Bulk modifications are particularly relevant to crystallinity, as they can generate new functionalities by altering intermolecular interactions [91].

Some studies on fructan modification have employed X-ray diffraction to investigate conformational changes before and after modification. Due to differences in fructan type and DP, the crystallinity results vary. Some studies quantified crystallinity using the crystallinity index, while others classified fructans as crystalline, amorphous, or semi-crystalline. Acetylated inulins have been reported to exhibit an amorphous state, in contrast to native inulin, which is crystalline [93]. However, in acylated agave fructans, a crystallinity index of 37.51–43.35% has been reported, which is slightly higher than of their native counterparts (23.88–34.46%), leading to their classification as semi-crystalline [42]. Unlike acetylation, acylation with fatty acids promotes the fructan crystallization. Fructans are inherently water-soluble and typically form amorphous structures due to their high degree of molecular branching [94]. This suggests that the effect on intermolecular interactions depends on the characteristics of the incorporated functional group, such as size, polarity, and others. To date, no other studies have reported the crystallinity of modified fructans.

## 4. Effect of Chemical Modifications on the Physicochemical and Functional Properties of Modified Fructans

This review highlighted the functional and biological benefits of fructan structural modifications. While fructans exhibit inherent bioactivity, their effects may be limited or insufficient for developing new applications. Structural modifications alter fructan conformation, enhancing their bioactivity by introducing new functional properties [95,96]. Therefore, assessing the impact of these modifications is crucial. Evaluating physicochemical and functional properties is essential, as they dictate fructan applications. The following sections discuss the physicochemical and functional properties assessed in studies on modified fructans.

### 4.1. Physicochemical Properties

After modification, it is crucial to evaluate the physicochemical properties of interest. For that purpose, some authors have evaluated certain properties, such as moisture, water activity, and color, among others. Moisture content is important because it determines the amount of total water present in the product; a low moisture content limits the water capacity and prevents or reduces agglomeration [97], while water activity provides information on the availability of water during degradation reactions [98]. Regarding color, its evaluation is imperative due to quality criteria. Color is dependent on physical characteristics, such as the shape or composition of the object being studied. The acylation of agave fructans with lauric acid showed a moisture content of about 7.0% and a water activity of 0.4. Thus, this chemical modification reduced the amount of OH groups, which were replaced by ester groups, inducing a lower hygroscopicity and thereby showing greater stability against enzymatic or microbial degradation [99]. Surprisingly, the color did not vary in luminosity and non-significant changes in its saturation were observed. The above is attributed to the incorporation of the fatty acid chain and the rearrangement of the structure [42]. Similar results were described in the acylation of agave fructan fractions with lauroyl chloride, obtaining lower moisture (3.42–6.45%) and water activity (0.10–0.31). It has been concluded that the fractionation of fructans (reduction of free sugars) and acylation contributed to the reduction in hygroscopicity to a greater extent. However, the color of these fructans was visibly brighter, making them attractive for incorporation into a product [7]. Comparatively, carboxymethylated fructans showed a water activity of 0.42, which is highly desirable since it has been reported that below this value, the plasticizing effect of water is kept to a minimum. Therefore, the mobility of the amorphous regions is restricted, keeping the fructans in powder form [70]. This modification, unlike acylation, incorporates hydrophilic groups, which increases the water-binding capacity of the fructan molecules. It is noteworthy that the results are strongly related to the physicochemical characteristics of the incorporated radicals, which lead to different behaviors in the fructans, which determine their functionality and application.

### 4.2. Functional Properties

It is essential to evaluate the impact of modifications on these fructan properties. This involves analyzing their enhancement or decrease, presence or absence, depending on the objective of the research work. Indeed, it has been reported that the acylation of agave fructans with fatty acids significantly decreased the solubility of the fructans due to the presence of OH groups. The introduction of fatty acid chains caused the formation of hydrophobic esters, providing hydrophobicity in the fructans. This hydrophobicity, in turn, increased the water retention capacity (0.01–5.75 g water/g sample), swelling capacity (0.10–94.57 mL/g), and oil (0.10–10.78 g oil/g sample). In addition, the high degree of substitution obtained (2.36) conferred new properties, such as foaming and emulsifying capacity. The increase in certain properties and the appearance of new ones are related to the amphiphilicity of the structure, which means that they are amphiphilic. This is due to the unique modification of OH groups and, thus, the replacement by ester groups. Therefore, the introduction of new functional groups promotes intermolecular interactions that are related to the chemical affinity of the polysaccharide [100]. In contrast, modified carboxymethylated fructans showed a swelling capacity of 0.80 mL/g and an insignificant oil retention of 3.99 g oil/g sample. It has been reported that the oil adsorption of polysaccharides is related to their chemical composition. Consequently, the carboxylate functional groups, being highly soluble in water, do not modify the fructans’ hydrophilic nature and thus keep their original properties [70]. Similarly, inulins, when esterified create amphiphilic polymers, which can form micellar aggregates in aqueous solutions above their critical concentration. These amphiphilic polymers can also allow the formulation of stable oil-in-water emulsions conferring great application potential in food, cosmetics, and pharmaceutical industries [45,46,59,61]. The modifications of fructans do not necessarily provide an enhancement of their functional properties since they depend on the characteristics of the implemented radicals that in turn determine their industrial application and scientific interest. For these reasons, it is noteworthy that not all studies focus on functional properties, but others lean towards prebiotic or biological activity, representing their main research objective.

## 5. Effect of Chemical Modifications on the Biological of Modified Fructans

The introduction of new functional groups into fructan molecules can modify their biological activity as well and confer new functionalities. Several studies have investigated the enhancement of specific biological activities in chemically modified fructans. For instance, studies have assessed the antioxidant, superoxide radical scavenging, and reducing capacities of carboxymethylated inulins. Carboxymethylation significantly enhanced these activities by introducing aminoheterocyclic residues and salts. As a result, these compounds demonstrated remarkable biological properties, including antimicrobial, anticancer, antiviral, anti-HIV, antisclerotic, antiparasitic, and antioxidant activities [101]. Therefore, combined modification strategies have been reported as feasible approaches to further enhancing biological activities, expanding their potential applications in biomedicine and other fields [69,71].

An in vitro test demonstrated a significant increase in antiproliferative activity when inulin-type fructans were modified with sulfate groups. Additionally, studies suggest that sulfation can significantly enhance the antitumor activity of fructans, depending on the concentration and incubation time [88,102]. Thus, sulfated fructans are promising candidates for anticancer applications [76].

Alternatively, the antimicrobial activity of acylated and succinylated agave fructans has been evaluated, showing significant inhibition (51–94%) against *Enterococcus faecalis*, *Salmonella typhi*, *Staphylococcus aureus*, and *Escherichia coli*. The effect exerted by each modification is attributed to the physicochemical properties of the introduced functional groups, which alter the structure and interactions of fructans. In acylated fructans, antimicrobial activity has been linked to byproducts formed during the degradation of long-chain fatty acids, which act as antimicrobial agents [103]. In particular, monolaurin, a degradation byproduct, destabilizes the bacteria cell membrane by increasing its permeability, ultimately leading to the cell’s death. Although polysaccharides alone possess intrinsic antimicrobial activity, acylation and succinylation further enhance this property [104,105].

Meanwhile, carboxymethylated agave fructans exhibited growth inhibition against *E. coli* and S. *typhimurium*, with effectiveness depending on the bacterial mechanisms and the concentration of the modified fructans. Differences in antimicrobial activity among carboxymethylated, succinylated, and acylated fructans may be influenced by variation in their structure, composition, and hydrophobicity [17,70].

Inulin derivatives modified by Schiff base reactions demonstrated high antifungal activity against phytopathogenic fungi such as *Botrytis cinerea*, *Fusarium oxysporum* f. sp. Cucumerium Owen, and *Phomopsis asparagi*. At a concentration of 0.1 mg/mL^−1^, the inhibition rates ranged from 54.4% to 94.0%. This antifungal activity was attributed to the lipophilic characteristics of the aromatic Schiff base, which facilitates the penetration of modified inulin into bacteria cells, leading to apoptosis. Additionally, modified inulin exhibited a hydroxyl radical scavenging rate of 100% [80].

Finally, acetylated inulin has also been reported, showing significant inhibition against the fungus *Bassiana* at a concentration of 5 mg/mL^−1^. These findings suggest its potential application in the development of microbiological control products, such as insecticides and microbicides [55].

This review highlights several significant benefits of modifying fructans with different functional groups. Each introduced group enhances the physicochemical properties of fructans while also conferring new chemical activities, ultimately broadening their potential applications.

## 6. Prospects and Challenges

Interest in inulin and agavin fructans continues to grow across various industries due to their remarkable physicochemical properties and health benefits. In recent years, chemical modifications have expanded their range of applications, demonstrating significant potential in multiple fields. It is now well established that fructans can undergo diverse modifications that enhance their physicochemical properties while introducing new biological activities. However, these modifications are influenced by several factors, including molecular structure, degree of branching, degree of polymerization (DP), degree of substitution (DS), solubility, and chain conformation.

Notably, the physicochemical properties of modified fructans are primarily determined by the functional groups introduced during the modification process. In some cases, the desired functionality or biological activity may need to be reassessed, as chemical modifications can lead to unexpected alteration in these properties. This review highlights several promising chemical methods that enhance the potential of fructans for the use as food ingredients, additives, and components in pharmaceutical, nutraceutical, biomedical and cosmetic products. Additionally, modified fructans hold promise in the development of prebiotics, symbiotics, and biological control agents. However, for these latter applications, further evidence is required to ensure safety, including comprehensive clinical and toxicity assessments, as well as deeper understanding of the underlying mechanisms.

While significant progress has been made in identifying new bioactivities and developing novel fructan characterization techniques, additional research is needed to elucidate the specific mechanisms underlying their biological functions. Despite numerous recent studies on the chemical modification and applications of fructans, further investigation is required before agave fructans (agavinas) can be scaled up for industrial applications. Future research should focus specifically on evaluating potential toxicity resulting from chemical modifications and gaining deeper insights into the structure–bioactivity relationship with stronger evidence.

Unlike inulin-type fructans, which have been extensively studied, agave-derived fructans are relatively less explored. However, their branched structure is of particular interest, as it may confer unique properties that make them highly suitable for food and pharmaceutical applications. Given their demonstrated potential, continued research on agave fructans is essential to unlock new opportunities for their commercial use.

## Figures and Tables

**Table 1:** Application and effects of modified inulin-type fructans.

Type of Modification	Improved Property	Observed Effect	Proposed Application	Reference(s)
Esterification (general)	Emulsifying capacity	Improved encapsulation and emulsion stabilization.	Food, cosmetic, and pharmaceutical industries	[45,46]
Acetylation	Enzymatic degradability	High encapsulation efficiency and targeted release	Encapsulation material for oral delivery	
	Antifungal activity	Inhibition of *Beauveria bassiana* at 5 mg/mL	Natural pesticide use	[55]
Succinylation	Hydrophobicity and modulated solubility	Encapsulation of antitumor drugs; release at neutral pH	Nutraceutical, medical, and pharmaceutical applications	[58,59,61]
Crosslinking (with PMDA)	Network density and pH sensitivity	Swelling decreases with increased crosslinking. pH-sensitive behavior	Modified hydrogels for colonic drug delivery	[66]
Schiff bases	Antifungal and antioxidant activity	Broad antifungal spectrum and free radical scavenging	Bioactive and biocompatible biomaterials	[80]
Carboxymethylation	Biocompatibility and bioactivity	High antioxidant and antibacterial capacity; enhanced antifungal effect via heterocycle modification	Medical, food, and cosmetic applications	[69,71]
Sulfation	Antiproliferative activity	Inhibition of tumor cell growth	Medical therapies with antitumor potential	[75]

**Table 2:** Functional effects of chemical modifications applied to agavin-type fructans.

Type of Modification	Improved Property	Observed Effect	Proposed Application	Reference(s)
Esterification	Hydrophobicity	Increased amphiphilic character; enhanced interaction with hydrophobic compounds	Food additives, emulsifiers, cosmetics, pharmaceuticals	[42,44]
		High drug encapsulation and bioavailability	Drug delivery systems for colonic release	[43]
Acetylation	Prebiotic activity	Improved survival of *L. paracasei*; fermentation by *S. boulardii*	Development of prebiotic and symbiotic formulations	[17,53]
	Hydrophobicity	Enhanced stability and prolonged drug release	Colonic drug transporters	[43]
Succinylation	Prebiotic and antibacterial activity	Enhanced bacterial viability and antimicrobial potential	Prebiotic, symbiotic, and antiseptic applications	[17]
Carboxymethylation	Thermal behavior and antimicrobial activity	Improved melting temperature; inhibition of Gram-negative bacteria	Functional food ingredients or bioactive additives	[70]

**Table 3:** Characteristic infrared absorption peaks in modified fructans.

Fructan/Modification	Characteristic Absorption Peak	Reference
Agavin/esterification	New signs at 2921 and 2847 cm^−1^ (CH_3_-CH_2_) and 1557 cm^−1^ (C=O)	[42]
	New signs at 2921 m^−1^ (CH_3_-CH_2_) and 1556, 1421 cm^−1^ (C=O)	[44]
	Increase in the intensity of bands a 2852 and 2923 cm^−1^ (CH_3_) and new signs at 1740 (C=O) and 12181187 cm^−1^ (C-O)	[43]
Agavin/acetylation	New signs at 1739.82 cm^−1^ (C=O) and 1368 cm^−1^ (CH_3_)	[17]
	New signs at 1700–1750 cm^−1^ (C=O)	[53]
	Increased signals at 1220, 1370 and 1740 cm^−1^ (CH_3_, C-O, C=O, respectively)	[43]
Inulin/acetylation	New peaks at 2900 cm^−1^ (CH_3_-CH_2_) and 1750 cm^−1^ (C=O)	[54]
	New peaks at 1745 cm^−1^ (C=O), 1370 cm^−1^ (C-H) and 1220 cm^−1^ (C-O)	[55]
Agavin/succinylation	New peaks at 2931 (OH) and 1643 cm^−1^ (C=O) that form the COOH and another sign at 1724 cm^−1^ (C=O) of the ester	[17]
	Two new peaks at 1576 (COO-) and 1734 cm^−1^ (C=O)	[59]
Inulin/crosslinking	New bands at 1729, and 1395 cm^−1^ (C=O), 1592 remains of COOH and 713 cm^−1^ aromatic ring (C-H) pyromellitic group	[66]
Agavina/carboxymethylation	New bands at 1596 and 1411 cm^−1^ (-COOH)	[70]
Inulin/carboxymethylation	New peaks at 1746 cm^−1^ and 1216 cm^−1^ COOH	[71]
Inulin/Schiff bases	Acetyl group at 1743 cm^−1^. New peak at 2107 cm^−1^ azido and other derivatives 1670–1680 cm^−1^ imine, 3060 and 1450–1600 cm^−1^ benzene ring	[80]
Inulin/sulfation	New peaks at 1250 cm^−1^ S=O, 814 cm^−1^ (C-O-S) and 3430 cm^−1^	[76]

**Table 4:** Characteristic 1H NMR signals in modified fructans.

Fructan/Modification	Sings NMR 1H (ppm)	Reference
Agavin/esterification	0.89, 2.65, 2.05, 2.19, 1.47, 1.28 methyl and methylene groups	[42]
	0.80, 1.25 methyl and methylene groups	[44]
	0.86, 1.2, 1.9 methyl and methylene groups	[43]
Agavin/acetylation	2.0 methyl group	[17]
	1.2 methyl group	[53]
	1.2 methyl and methylene groups	[43]
Inulin/acetylation	2.03 acetate group	[54]
Agavin/succinylation	1.91 methylene group	[17]
	1.26 and 1.94 methyl and methylene groups	[59]
Inulin/carboxymethylation	4.51 methylene groups	[71]
Inulin/Schiff bases	2.0 hydrogen of the acetyl, 3.7 H–C6–N, 8.4 H–C7=N and 6.5–8.0 aromatic rings	[80]

## Data Availability

No new data were created or analyzed in this study. Data sharing is not applicable to this article.
